# *CyanoHAB* Occurrence and Water Irrigation Cyanotoxin Contamination: Ecological Impacts and Potential Health Risks 

**DOI:** 10.3390/toxins1020113

**Published:** 2009-11-25

**Authors:** Sana Saqrane, Brahim Oudra

**Affiliations:** Laboratory of Biology and Biotechnology of Microorganisms, Microbiology and Toxicology Environmental Unit, Faculty of Sciences Semlalia, University Cadi Ayyad, B.P. 2390, Marrakesh 40 000, Morocco; Email: saqranesanaa@yahoo.fr (S.S.)

**Keywords:** crop plants, *CyanoHAB*, health hazard, microcystins (MCs), phytotoxicity, water irrigation

## Abstract

The world-wide occurrence of harmful cyanobacteria blooms “*CyanoHAB*” in fresh and brackish waters creates problems for all life forms. During *CyanoHAB* events, toxic cyanobacteria produce cyanotoxins at high levels that can cause chronic and sub-chronic toxicities to animals, plants and humans. Cyanotoxicity in eukaryotes has been mainly focused on animals, but during these last years, data, related to cyanotoxin (mainly microcystins, MCs) impact on both aquatic and terrestrials crop plants irrigated by water containing these toxins, have become more and more available. This last cited fact is gaining importance since plants could in a direct or indirect manner contribute to cyanotoxin transfer through the food chain, and thus constitute a potent health risk source. The use of this contaminated irrigation water can also have an economical impact which appears by a reduction of the germination rate of seeds, and alteration of the quality and the productivity of crop plants. The main objective of this work was to discuss the eventual phytotoxicity of cyanotoxins (microcystins) as the major agricultural impacts induced by the use of contaminated water for plant irrigation. These investigations confirm the harmful effects (ecological, eco-physiological, socio-economical and sanitary risk) of dissolved MCs on agricultural plants. Thus, cyanotoxin phytotoxicity strongly suggests a need for the surveillance of *CyanoHAB* and the monitoring of water irrigation quality as well as for drinking water.

## 1. Introduction

**Figure 1:**
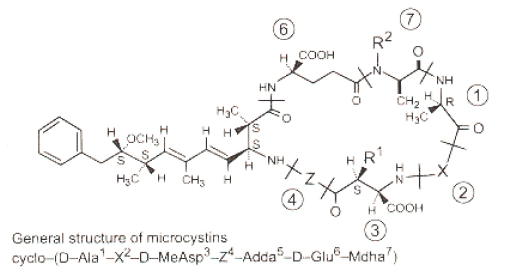
General structure of microcystins (cyclic heptapetides hepatotoxins) [7].

Cyanobacteria (blue-green algae) are photosynthetic prokaryotes which frequently form harmful blooms in eutrophic water bodies (*CyanoHAB*). Some species of cyanobacteria are able to produce toxins (cyanotoxins) which may be divided into three main groups; hepatotoxins, neurotoxins, and cytotoxins [1]. Microcystins (MCs), part of the hepatotoxin group, are the most world-widely distributed cyanotoxins produced by several algal genera, including *Microcystis, Anabaena, Oscillatoria,* and *Nostoc* [2]. Microcystins are cyclic heptapeptides with the general structure cyclo(-d-Ala-l-X-erythro-b-methyl-d-isoAspl-Y-Adda-d-isoGlu-*N*-methyldehydro-Ala) (Figure 1). The aminoacid Adda (3-amino-9-methoxy-2,6,8-trimethyl-10-phenyldeca-4,6-dienoic acid) is considered responsible for the molecules’ hepatotoxicity [3]. Microcystin-LR (MC-LR) is one of the predominant variant of MCs produced by cyanobacteria blooms [4]. 

Their toxicities resulted from the inhibition of the catalytic units of serine/threonine protein phosphatases types 1 (PP-1), 2A (PP-2A), and 3 (PP-3) [4]. On the other hand, recent studies have also reported that MC-LR and NOD-R induced oxidative stress [6]. These toxins can cause water quality problems for fisheries, aquaculture, farming, and sanitary hazards for human and animals. Therefore, surveys of *CyanoHAB* and cyanotoxins have been carried out in several countries, aimed at avoiding sanitary risks by a safe use of cyanotoxin-contaminated water [7]. However, in water irrigation, cyanotoxin surveys have not yet been considered within any water quality official monitoring program, in spite of the possible contamination of edible plants constituting a significant indirect route of human exposure to cyanobacterial toxins [1]. Accordingly, many studies clearly indicate that irrigation with water containing cyanotoxins can be a threat for both the quality and yield of crop plants [8,9]. Also, the confirmed bio-accumulation of these toxins in plant tissues [8,9] constitutes a route for introduction of these toxins into the human food chain and ultimately a human health concern [9,10]. Ko´s *et al*. [11] reported for the first time that MC inhibited the growth of mustard seedlings. Since then, the research interest on phytotoxic effects of cyanotoxins on terrestrial plants has increased, showing physiological and morphological alterations by cyanotoxins in a range of terrestrial plants [12,13]. 

Although rare, the results of laboratory work have highlighted the harmful effects of cyanobacteria aqueous extracts on the growth and the development of the seedlings, which could be regarded as simulation of the chronic exposure of these plants to the cyanotoxins conveyed by water irrigation. In this review, we will examine and discuss the problems related to the use of irrigation water that does not conform with the hygiene standards relating to the particular contamination by toxins of cyanobacteria. As recommended by WHO, cyanobacterial toxin contamination should be taken into account for drinking and bathing waters [7]; with this review we hope to start the debate around the contamination of water irrigation by the cyanotoxins, as well as, in the case of drinking, recreational and bathing waters, so the focus of this review will be especially on the eco-physiological and economical effects and sanitary risks potentially caused by irrigation with water containing toxic cyanobacteria and cyanotoxins. 

## 2. Ecological and Physiological Effects

### 2.1. Effects of cyanotoxins on seeds germination rate

Recently, the effects of cyanotoxins on seed germination and seedling growth has attracted the interest of researchers because it proved that use of water contaminated with cyanotoxins in irrigation contribute to and explain observed lower field production [9,14]. In this way several terrestrial plants were investigated using both cyanobacterial standard toxins and cyanobacteria cell-free crude extracts. 

It appears that exposure to cyanobacteria aqueous extract may affect the metabolic activities of seeds during the germination process. It is important to point out that some tested plants such as .. *esculenta*, are more resistant to MC extract exposure. This resistance could be explained by a detoxification process, mediated by enzymatic activity, as described for *Lepidium sativum* seedlings [15]. Similar results previously were reported by Chen *et al*. [12], using rice (*Oryza sativa* L.) and rape (*Brassica napus* L.) seeds. Microcystins had more powerful inhibition effects, measured as germination percentage of seeds and seedling height, on rape than rice. After 10 days of exposure, higher toxin levels (0.6 µg/mL) caused significant differences in germination percentage of rape seeds and no normal seedlings were observed at a concentration of 3 µg/mL since rape seedlings with more than 50% necrosis to leaf tissue generally cannot be regarded as standard seedlings [12]. Inhibition of germination was also observed after the exposure of alfalfa (*Medicago sativa*) seedlings to cyanobacterial toxins (Microcystins and Anatoxin-a) and cyanobacterial cell-free crude extract [14]. All these investigations suggest that exposure to MCs via water irrigation contaminated with toxic cyanobacteria can pose a threat to the quality and yield of crop plants in the agricultural environment. Chronic plant exposure to MCs could have a net repercussion on the life cycle of the plants. 

Exposure during four days to cyanobacteria aqueous extract containing MC-LR has induced a significant effect on the reduction of germination rate of terrestrial plant seeds such as *Lens esculenta, Zea mays, Triticum durum* and *Pisum sativum* [16]. However, the effect of MC on seed germination rate was dose dependent and significantly different according to the sensitivity of these tested plants. *Lens esculenta* seeds appear to be the most resistant ones, with the maximum germination rate of 74.67%. *Zea mays, Triticum durum* and *Pisum sativum* showed germination rates of 71.33%, 40.67% and 3.00% at 11.6 µg.mL^-1^, respectively. There is evidence that aqueous extract containing MC-LR inhibits strongly the germination of *Pisum sativum* seeds, which seems to be the most sensitive species among the four tested [16]

### 2.2. Effects of cyanotoxins on seedlings growth and development

In addition to the inhibitory effect on seed germination, cyanotoxins could also have a negative impact on the growth and development of the seedlings. These effects have previously been reported by several authors [9,11,14,17,18]. In most cases, effects on growth and leave and root development were recorded. Thus, Microcystin-RR at 0.005 µg/mL caused malformations in white mustard (*Sinapis alba*) seedlings, reduced seedling weights and lateral root formation, and inhibited protein phosphatase 1 and 2A [17]. Growth of potato (*Solanum tuberosum*) cultures were reduced at 0.005 mg/kg MC-LR in the a solid culture medium, and completely inhibited at 0.5-5 mg/kg MC-LR, while growth of bean (*Phaseolus vulgaris*) plants in culture was inhibited by MC-LR at 1.12 mg/kg [18]. Gehringer *et al*. [15] observed a significant decrease in leaf and root lengths and in productivity of *Lepidium sativum* seedlings caused by cyanobacteria MC-extract. Pure MC-LR caused a similar decrease, indicating that the effect of the cyanobacteria extract was due to the MCs. Later, it was reported that different spinach variants were all morphologically affected after 6-week exposure to a cyanobacteria crude extract containing 0.5 µg/mL MC-LR [13]. Additionally, this study showed that different variants of the same plant species can react in different ways to the same toxicant. Plants could be damaged by the toxins [14] and growth inhibition occur, which will then lead to a decrease in crop yield. This hypothesis has been recently confirmed by Saqrane *et al*. [10], who studied 30-day exposure of *Triticum durum*, *Zea mays*, *Pisum sativum* and *Lens sculenta* cultivars to cyanobacteria extract containing 0.5-4.2 mg equivalent MC-LR/mL. A net reduction in height plant, as well as in the leaf number and root length were observed. Plant yield, determined by either fresh or dry biomass, was also decreased [10]. With respect to seedling root development, the total growth inhibition of the lateral roots observed on *Pisum sativum* was briefly explained by Saqrane *et al*. [16] (see Section 2.3). 

### 2.3. Cytological modifications

According to Saqrane *et al*. [16], exposure to cyanobacteria extract containing MC-LR induces a net histological modification of the primary root tissue of *Pisum sativum* seedlings. These cytological modifications were evidenced by a delay in the organ root differentiation and formation of vascular cylinder and inhibition of lateral root primordial formation. All these histological anomalies could explain the negative impact on the growth and development of plant roots caused by microcystin exposure. In comparison with controls, the root cuts exposed to cyanobacteria extract indicated the absence of endodermis and peri-cycle cells, and there is no lateral root primordial formation. Also, a delay in the formation of primary xylem and the absence of fibers was observed [16].

### 2.4. Photosynthetic activity and chlorophyll content

The chlorophyll content is a general indicator of a plant’s condition. Thus, impaired photosynthesis of terrestrial plants by cyanobacterial toxins has been detected in many species. Chlorophyll content of potato (*Solanum tuberosum*) cultures was reduced at 0.005 mg/kg MC-LR in the culture medium [18]. Cyanobacteria aqueous extract containing various MC variants caused a significant decrease in chlorophyll (*a* + *b*) content in *Z. mays* and *L. esculenta* following 30-day exposure to 2.1 and 4.2 µg/mL, with the decrease respect to the untreated plants being 22-25%, but no significant effect on *T. durum* and *P. sativum* was observed [10]. The physiological state of the photosynthetic apparatus was determined using the Fv/Fm ratio of the fluorescence measurements. Values of Fv/Fm lower than 0.8 will be seen when the plant has been exposed to stress, indicating in particular a photo-inhibition phenomenon [19]. In the four tested plants *Triticum durum*, *Zea mays*, *Pisum sativum* and *Lens sculenta*, and at the various MC concentrations (0.5-4.2 μg equivalent MC-LR mL^-1^), cyanobacteria extract treatment produced a decrease in the Fv/Fm ratio. The Fv/Fm decline was dose and plant species-dependent, with the most sensitive species being *P. sativum*, in which the Fv/Fm reduction produced at 1 µg/ mL was around 70%. Fv/Fm decrease clearly indicates that MC impaired the photosynthetic activity of all exposed plants, especially of *P. sativum*, and that the impairment was at least partially due to a damage of PSII reaction centers [10]. In another study, watering broccoli (*Brassica oleraceae* var. *italica*) and white mustard (*Sinapis alba*) seedlings irrigated with water containing 0.001 or 0.01 µg/mL microcystins had no effect on the concentrations and relative ratios of the photosynthetic pigments chlorophyll . and chlorophyll .. [20]. The values of fluorescence in broccoli were all within the range of 0.82-0.85, which is typical for healthy plants [21]. For mustard the photosynthetic apparatus was mildly damaged. The value of Fv/Fm ratio for the control plants was, however, also low, indicating that the observed damage could not be ascribed to toxin exposure [20]. 

### 2.5. Oxidative stress

As a result of exposure of both animal and vegetal cells to toxicant (microcystin-like), reactive oxygen species (ROS) can be generated [22,23], which react with other cellular compounds such as lipids, proteins and DNA [24,25,26]. The well elaborated antioxidative defence system of plants functions to relieve the negative effects caused by reactive oxygen species. This antioxidative network consists of enzymes, such as superoxide dismutase (SOD), catalase (CAT) and ascorbate peroxidase, and also nonenzymatic antioxidants, such as reduced glutathione and vitamins [27,28]. The promotion of oxidative stress due to exposures to cyanobacterial toxins was shown in different plants from aquatic macrophytes [23,29] up to higher terrestrial plants like *Brassica napus*, *Oryza sativa* and *Medicago sativa* [12,14]. More important would be to investigate in terrestrial plants the possibility of forming stable bound residues with cell wall components. Gehringer *et al*. [15] measured an increase of glutathione peroxidase (GP-X) activity after exposure of *Lepidium sativum* seedling to MC-LR, indicating an oxidative stress response. Lipid peroxidation (LPO) is a possible damage caused by increased amount of ROS due to exposure to cyanobacterial toxins in plants. In this study LPO, as sign for the damage due to the enhanced generation of ROS, was measured after exposure of *L. sativum* seedlings to MC-LR and toxic cyanobacterial crude extract. At present, there is some evidence suggesting that oxidative stress might be involved in the toxicity of microcystins on aquatic plants [30]. 

In accordance with the suggestion that oxidative stresses are involved in the toxicity of microcystins to plants, it has been reported that the activity of peroxidase (POD) and superoxide dismutase (SOD), two antioxidant enzymes, were changed in *Brassica napus* L. and *Oryza sativa* L. seedlings after exposure to microcystins [12]. Also, oxidative damage, such as lipid peroxidation, was detected after the exposure of *Medicago sativa* seedlings to the toxin. Reactive oxygen detoxifying enzymes were elevated, showing a marked response in *Medicago sativa* to oxidative stress caused by the exposure to cyanobacterial metabolites that might influence the growth and development of these plants negatively [14]. The elevation of antioxidative enzymes in spinach after six weeks of exposure clearly indicates that oxidative stress is promoted in plants by exposure to a cyanobacterial crude extract containing MC-LR [13]. The transcript levels of copper/zinc (Cu/Zn) SOD (E.C. 1.15.1.1) isoforms primarily found in chloroplasts and in the cytosol of plant cells [31] were compared in the spinach variants Sharan and Matador. An accumulation of mRNA was found in Sharan after exposure to the crude extract. In the variant Matador, both cytosolic and chloroplastic SODs were induced. The increased level of chloroplastic Cu/Zn SOD might lead to a higher level of H_2_O_2_, which is the end product of the SOD reaction. Hydrogen peroxide could then, if not quickly removed, react with the superoxide anion radical to form the highly toxic hydroxyl radical [32], which might inhibit photosynthesis, and could explain the inhibition of photosynthesis as observed in the spinach variants exposed to microcystin [13]. In addition, the induction of Cu/Zn SOD at the molecular level is closely correlated with the concomitant synthesis of the corresponding protein, measured for example as specific SOD enzyme activity. Other enzymes involved in antioxidative defence, such as the glutathione *S*-transferase (GST) system and GR, showed a clear increase in activity, as was also found by Gehringer *et al*. [15] and Pflugmacher *et al*. [14]. On the one hand, the GST system conjugates and detoxifies the metabolites of lipid peroxidation and in addition is the key enzyme for the biotransformation of microcystins to a glutathione conjugate [33,34], whereas, on the other hand, glutathione reductase (GR) is certainly necessary to maintain the glutathione pool, as was also shown by [35] and [36]. Because an increase in GSH was detected in most exposed spinach variants, with the exception of the variant Ballat, this might be explained as a long-term adaptation mechanism to toxin exposure. 

## 3. Potent Sanitary Risk

Uptake of cyanobacterial toxins by agricultural plants could occur via spray irrigation of crop plants when surface water containing cyanobacteria is used. The uptake of these toxins by agricultural plants poses a potential human health risk when they enter the food chain [17,18,37]. Thus, several authors have shown that terrestrial plants are able to accumulate MC in sufficient concentration to induce morphological and physiological changes [12,14,15]. Recently, Peuthert *et al*. [8] reported the uptake of MC-LR and MC-LF by seedling roots of 11 agricultural plants, and their translocation to shoots. Spray irrigation of commercial lettuce (*Lactuca sativa*) plants with water containing *Microcystis* resulted in colonies and single cells of the cyanobacterium being lodged on the leaves 10 days after the last irrigation [37]. MC-LR was present at 2.5 mg/kg dry weight (DW) in the central leaves, 0.833 mg/kg (DW) in the distal zone of mature leaves, and 0.094 mg/kg (DW) in the basal zone of mature leaves. The study indicated that toxins were absorbed by the plants as the central leaves would have been protected from irrigation. The cyanobacterial cells were not removed by washing the leaves in water. The authors recommended investigation of the fate of cyanobacterial cells and toxins during and after spray irrigation with water containing cyanobacteria, to contribute to development of policies on the use of such water and the acceptability of plants for human consumption after irrigation with contaminated water. Similar conclusions were reached for *Oryza sativa* and *Brassica napus* [12]. They suggest that consumption of edible plants exposed to microcystins via irrigation may have health risks. Greater quantities of microcystins were recovered from the shoots of *Brassica napus* than from *Oryza sativa*, indicating that different plant species may accumulate microcystins at different rates. 

Evidence of MC translocation within the plant is scarcely documented, with the available reports on seedlings of other agricultural plants, e.g., *Medicago sativa* L., *Triticum aestivum* L., *Cicer arietinum* L. [12], *Brassica oleracea* var. *italica* and *Sinapis alba* [20] being very recent. Also, Cruch *et al*. [9] reported the accumulation and translocation of cyanotoxins in three forages irrigated with lake water containing microcystins. For the shoot application treatment, microcystins were not present at measurable levels in shoots of ryegrass or rape, but were present in lettuce [0.79 mg/kg dry weight (DW)] and clover (0.20 mg/kg DW). Total microcystin concentration in roots did not vary greatly depending on whether treatment water was applied directly to the sand, or reached the roots via run-off from the shoots. Microcystins in roots were highest in clover (1.45 mg/kg DW), intermediate in lettuce (0.68 mg/kg DW) and low in ryegrass (0.20 mg/kg DW), and rape (0.12 mg/kg DW). There was no evidence for root-to-shoot translocation of microcystins. The results show that irrigation with water containing microcystins has the potential to move microcystins into farm animal and human food chains at concentrations that can exceed recommended tolerable limits. In none of those reports, a continuous assay of the different MC variants present in the various plant parts was carried out. However, Saqrane *et al*. [10] analysed quantitatively and qualitatively the MC variants accumulated in different organs of *Triticum durum, Zea mays, Pisum sativum* and *Lens esculenta.* In this study it was determined the MC variant percentages in roots, stem and leaves for each plant. Considering the relative abundance of each MC in the cyanobacteria extract and the amount of MC content in each organ, the MC of choice for uptake were: MC-YR in *T.durum*, MC-LR and MC-(H4)YR in *Z. Mays*, MC-FR and MC-RR in *P. sativum*, MC-RR in *L. esculenta*. In each exposed plant, MC variants were taken hydrophobicity-dependently. MC-RR (40 to 70%) was more taken up than MC-FR (4 to 20%) and WR (2 to 16%). From the health risk point of view, special attention should be paid to important topics such as MC allocation in the plant, degree of accumulation and toxicity of the diverse MCs, as well as possible relation among different cyanobacteria components, which may lead to synergistic and antagonistic effects.

## 4. Conclusions

To conclude, all realised studies support the idea that first, the use of surface water containing MC for irrigation can affect both plant crop yield and quality, and second, that MC accumulation in edible plants could pose a potential risk for human and animal health, if the MC intake exceeded the recommended tolerable limits. In general, these cited investigations references confirm the harmful effects (ecological, eco-physiological, socio-economical and sanitary risk) of dissolved MCs on agricultural plants. Thus, cyanotoxin phytotoxicity highlights the need for the surveillance of *CyanoHAB* and the monitoring of water irrigation quality as well as for drinking water.
